# Supplementary material for: Individual and community-level factors associated with animal source food consumption among children aged 6-23 months in Ethiopia: Multilevel mixed effects logistic regression model
Source: PLoS One. 2022 Apr 5;17(4):e0265899. doi: 10.1371/journal.pone.0265899 (PMC8982870; doi:10.1371/journal.pone.0265899)
Supplement: Attachment — Submitted filename: Review Abdu et al..docx [file pone.0265899.s001.docx]

Line 23: The background section could be strengthened by a) emphasizing the importance of ASF (rather than diversified diet), b) emphasizing the age group.

Line 27: Suggest changing ‘multi-layered’ to more specific – perhaps individual and community level.

Line 30: Sentence beginning with “a stratified two-stage” is unclear. What is complex sample design referring to? How were weights used?

Line 38: I’m curious about the inclusion of home delivered children as a variable, as it may have substantial overlap with some of the other predictors, particularly low SES. In addition, what overlap is there between low SES and high community poverty level, outside using it as both an individual and community level factor?

Line 47: I would love to see the conclusions be a bit more specific in terms of what targeting and policy decisions could be incorporated based on the findings.

Line 62: Suggest re-arranging the background to begin with animal-source foods role in optimal development, rather than the indicator, which belongs later or in the methods section.

Line 71: There are also several more recent publications and meta-analyses which discuss the importance of ASF – inclusion of those would strengthen the background section, which relies on several single-sited analyses or position papers. Please see <https://academic.oup.com/advances/article/10/5/827/5513046?login=true> and <https://www.cochranelibrary.com/cdsr/doi/10.1002/14651858.CD012818.pub2/full>.

Line 76: Suggest “widespread” livestock production, or something similar

Line 78: Suggest revising the line.

Line 85: Curious if ASF should be considered a practice? Practice to me would suggest a cultural component, but I believe what this analysis is getting at is its availability, affordability, accessibility, etc. Does not necessititate a revision if practice is what is intended – just some elaboration.

Line 93: Language is a bit informal

Line 98: It would be helpful to discuss why high-risk fertility behaviors is specifically highlighted here.

Line 105: No need to include the names of variables in this section.

Line 120: It would be helpful to explain why the study used children ages 6-23 months in particular. This may be better included in the background where the importance of ASF in this age range is emphasized – otherwise there is an equally strong argument to use a wider age range.

Line 127-130: I’m curious why Group 4 dairy products isn’t considered here, as this is also an animal source food. This would necessitate re-running all analyses, however unless there is a strong theoretical reason for excluding it, it should also be included.

Line 130: It’s important to note that this indicator does not exactly refer to adequate ASF consumption, but that ASF consumption is necessary for minimum dietary diversity. It’s therefore inaccurate to say “adequate animal source food consumption,” as this is only a criterion for MDD. I would simply rephrase to ASF consumption or no ASF consumption, but avoid labeling the variable as adequate, since there is technically no indicator for this.

Line 135: It would be helpful to include citations that support the use of ‘high risk fertility behaviors’ from a theoretical perspective. I can understand why mother’s age <18 might influence ASF consumption as a proxy for poverty or decision-making, but not mother’s age >34 or birth order above three. These are more adequately capturing risks to the mother and child during birth and early life but I don’t see the connection to ASF consumption.

You might also consider incorporating some of the variables in the DHS that relate to household decision-making. Women’s decision-making power has been shown to influence infant and young child feeding – those would seem to have more of a theoretical basis than the fertility behaviors.

Line 148: Don’t need to include Stata commands or variable names throughout this or later sections. I’m curious here about the reasons for using community poverty level and community education level, as I don’t think they’re well explained. You might also note here that

Line 250: I’ve discussed inclusion of different variables in several other sections but also want to highlight a few more that seem missing. In particular, I believe DHS asks whether households own any livestock, which would be important to include. I apologize if I am wrong on that.

Line 273: I’m curious why variables with a p-value of <0.25 were included – you may want to include a citation for this. Otherwise, I would suggest revising many of these analyses so that there is a strong theoretical basis for their inclusion. Since the paper is interested in exploring variables that are associated with ASF and designing policy around that, it should be made clear why, for example, ANC visits or place of delivery are useful predictors of ASF consumption. The same holds true of fertility risk.

Line 280-284: I would like to see in an appendix any additional model fitting that you carried out with different variables. As stated above, many of these variables don’t seem to have a strong rationale for inclusion. Model fit generally increases with more variables, but it would be important to demonstrate by how much, precisely, to justify their inclusion. You note tat the very end that associations with a p-value below 5% were included, but in what stage of the process did you fit the variables?

In particular, I would be interested to know whether inclusion of community level variables is was even warranted. You present AIC and DIC values for the null model and final model, but showing AIC values of various iterations of models would be more helpful. A very good overview for fitting multi-level models is here: <https://www.douglasluke.com/book/mlm/> - I would pay particular attention to assessing model fit through analysis of variance of the models detailed in Chapter 3.

I would also use this as a guide in assessing model fit via inclusion of the variables that seem to overlap with each other – for example ANC visits and poverty/access to healthcare, etc.

Finally, did you explore any interactions between these variables? My overall comment would be to provide substantially greater justification and details on how you conducted the model building process.

Line 284: Correct o p-value below 0.05.

Line 328: It would be helpful to re-organize this table to separate individual and community level factors more clearly.

Line 365: This statistic comes from one survey in Indonesia, which should either be noted, or the statistic removed.

Line 358-370: This discussion would be better used to present more of the overall findings from your analysis – rather than other analyses. This discussion only mentions that ASF consumption is low and more prevalent in food insecure regions. It should be connected back to the main themes from your findings.

Line 416: This is a great explanation of possible pathways for ANC visits, though I would be curious whether these variables significantly improve model fit individual when included with education and wealth. If so, this is a great finding, but I would also like to see it explained more – e.g. what messages do mothers receive? Finally, is there a reason for including both ANC visits and home birth?
