# Supplementary material for: Individual and community-level factors associated with animal source food consumption among children aged 6-23 months in Ethiopia: Multilevel mixed effects logistic regression model
Source: PLoS One. 2022 Apr 5;17(4):e0265899. doi: 10.1371/journal.pone.0265899 (PMC8982870; doi:10.1371/journal.pone.0265899)
Supplement: Attachment — Submitted filename: TA review comments- PONE-D-21-25620.docx [file pone.0265899.s002.docx]

Review comments for:

Individual and community-level factors associated with animal source food consumption among children aged 6-23 months in Ethiopia: Multilevel mixed effects logistic regression model.

This is a very good piece of work. I have enjoyed reading it. The topic has important policy implications, the method suits the nature of the data structure, and the effort put into the manuscript to make a robust explanation is admirable. My general feedback is very good with mainly one editorial/additional estimation correction required apart from the routine editorial details I found. For this main comment, please see 2.1. Other than this, the followings are some of the comments I put forward in each subheading:

1. **General comments**
   1.1. “Animal source food consumption is very low” (line 47) – reference? Compared to?
   1.2. “despite a better livestock population, diversified diet consumption is poor..” should it be diversified ASF? ( line 76)
   1.3. Which time period are the data points referring? (line 78)
   1.4. “So far, limited attempts were tried to understand the problem with the use of small-scale surveys, despite the multifaceted nature of the issue, where individual or household level factors only may not be sufficient” (line 86 – 88). What are these studies? A few citation of these studies will be ideal. How is your study unique from these studies?
   1.5. “Nationwide study addressing the factors that affect the level of ASF consumption has not been conducted in Ethiopia” (line 97 – 98) I am afraid I can’t agree to this claim. For example, *Workicho et.al (2016) Household dietary diversity and Animal Source Food consumption in Ethiopia: evidence from the 2011 Welfare Monitoring Survey*, *Potts et.al (2019) Animal Source Food Consumption in Young Children from Four Regions of Ethiopia: Association with Religion, Livelihood, and Participation in the Productive Safety Net Program* are a few examples. You can cite these researchers and point out what makes your study unique.
   1.6. “Currently, the price of ASF is rising alarmingly in the country above 36%”. Lin 381. This should be re-written. There is no single ASF price as such unless it is a price index, I think.
   1.7. “In Ethiopia, 35% [37], 57% and 36.8% [9] are a victims of Zinc deficiency, anemia and stunting…”line 438, who are these figures referring to? Of the Ethiopian population, Ethiopian children or ?
2. **Methodology**

Measurement of the dependent variable – while a dichotomized variable is a good starting point, it is also crude as it aggregates measurement and somehow undermines heterogeneity, for example in the type of ASF consumed here. In this regard, as a robustness extension,

2.1. In addition, “A large scale regional variation (..) in Addis Ababa and … lowest in Somali was observed in ASF consumption aged 6-23 months.” (line 262 – 264). And “Low ASF consumption is more prevalent in more food insecure regions of the country (Afar, Somali, and other regions).” (Line 361 – 362). “Apart from the resource scarcity related factors, one of the reasons for this disparity could be measurement of ASF, as we do not considered milk and milk products widely used in pastoralist communities of Ethiopia”.

This provides a wrong information and introduces systematic bias. Afar and Somali constitute one of the largest livestock population making children more likely to consume milk and milk products. These regions do not have similar wealth of chicken and are less likely to consume egg.

As a result, either

- Bring the definition of ASF used in this manuscript forward explicitly at the start (Mainly in the dependent variable section) and give a concerted direction to your reader what you mean by an ASF or
- Include milk and milk products and do additional analysis,
- Leave Afarand Somali from your analysis. My advice for you is to include milk and milk products and re do the analysis while keeping the current results as they are and see how things change. This will give you percentage values of ASF consumption and regression estimates to which you can compare results.
  2.2. Will a separate regression (as a dichotomous variable) be an extension of this study or be used as a robustness check? For example, estimating the same model for the consumption of Egg and other flesh foods alone.
  2.3. Counting the number of ASF and generating an ordered variable to run multi-level ordered logistic regression provide similar results?
  2.4. “Only 27 (0.6%) of mothers had accesses to all three media at least once a week” which three media? I think this needs re-phrasing the statement. Looking at Table 1, this variable does not show variability worth to have it in a description. 99% said No to media access. This requires re categorization or will not make a good predictor. This is also true for “community level education” where only 5% are in the high education group. (Table 1)
  2.5. Table 2 could give more information if the second row is subdivided into each type of flesh foods. The first row gives the aggregate measure already.

3. **Results and discussion**
   3.1. “The finding of this study revealed that children whose mothers had at least four ANC visit and gave birth at health facility had a protective effects against low ASF consumption. The possible pathways (mechanisms) of this association may be due to ease of access to health information that may improve the likelihood of mothers’ practice of proper child feeding” (line 415 – 418). Can you provide evidence to this in your research, please?
   3.2. “The current analysis indicated that the ASF consumption of children in Ethiopia is 22.7%” – better if written as “Currently, only 23% of children in Ethiopia consume ASF” of something similar along this line otherwise, the conclusion drawn from this will be over riding.
   3.3. Policy implication scattered here and there. They should be brought together under one heading or in one or two paragraphs. For example, line 381 to 388 against lines 442 to 445
