# Supplementary material for: Individual and community-level factors associated with animal source food consumption among children aged 6-23 months in Ethiopia: Multilevel mixed effects logistic regression model
Source: PLoS One. 2022 Apr 5;17(4):e0265899. doi: 10.1371/journal.pone.0265899 (PMC8982870; doi:10.1371/journal.pone.0265899)
Supplement: Attachment — Submitted filename: Response to Reviewers.docx [file pone.0265899.s004.docx]

From authors

Manuscript title: Individual and community-level factors associated with animal source food consumption among children aged 6-23 months in Ethiopia: Multilevel mixed effects logistic regression model

Manuscript ID: PONE-D-21-25620R1

Dear editor and reviewers

I am very grateful for all valuable comments and suggestions you gave us for the improvement of the paper. Hereunder, I responded to each point raised in the second-round review and these amendments are indicated in track change in the revised manuscript when applicable and clean copy of the final manuscript is submitted.

**Editor’s comments**

#1 The issue regarding depositing the laboratory protocol in a relevant repository, since all methodological approaches are stated and indicated in the manuscript, it is not applicable for this case.

It is not applicable

#2 We did not make any change to financial disclosure

\# 3 the issue of retracted references

Here we used the scite tool to check for retracted references and w updated the reference section accordingly. We also manually checked for retracted references and citation with retracted referencing were managed accordingly.

**Reviewers’ comments**

Fortunately, almost all the comments have been addressed in the first revision and editor and the reviewers duly acknowledged our effort, thanks for that. But there are some comments that may need clarification from the authors. First of all, we are glad to have your valuable comments.

Reviewer 1 comments

#1 on the introduction

Response; we accept the comments and we included other robust and more comprehensive evidence on the issue. The references are updated in the revised version.

#2 Reword table 3

Response: It is revised as requested “no ASF consumption”.

#3 the issue of Milk is different from ASF

Response: in the previous revision, we clearly indicated the definition of the indicators and ASF is defined in WHO in a way we define it in the current paper. But I accept, the issue to include correct references to the indictor definition. In order to be in line with the WHO updated new indicators, we still stand to define ASF consumption (as indicator)-as egg and/or flesh consumption [1] as such food groups are different from milk consumption among children due to

#1 majority of children consume breast milk up to two years

\# 2 proportion of older children who consumer milk (cow, goat, camel) is high but,

#3 the typical consumption of egg or flesh meats has a paramount importance where access to such a food is low in developing countries due to rising price of animal source foods. Moreover, such foods are rich source of iron, zinc, vitamins and other mineral necessary for optimal growth in addition to protein, carbohydrates and fats which are common in milk. But the first three nutrients are found in scant amount from milk and milk products.

It is due to the above evidences that WHO excludes milk and milk product from the definition. I hope you will understand that.

#4 high risky fertility behaviors

Response: above all the point raised by the reviewer here is strong and logical to us to reconsider the decision regarding variable selection. The reason we included this variable in the model is due to the following.

A. The inclusion of this variable greatly improved the model fitness and it is found in many different literatures that High fertility behavior is strongly linked to food security, access to nutritious food and nutritional status of children (stunting).

We can clearly understand from the pooled analysis of DHS surveys from 2006 to 2012 that fertility behavior is important factor that determine access to food and nutritional status of children [2].

B. as this indicator shows the overall fertility risk of mother of children, this may indirectly indicate or correlate with birth interval and number of children, which in turn affects the ASF consumption negatively, we included it in the description part.

#5 copy edition and proofreads

The revised manuscript undergoes a thorough copy edition and proof read by professional editor as indicated in the revised manuscript (when applicable) and cleaned manuscript.

Reviewer 2 comments

-the reviewer is convinced by the revisions made and the compelling rational arguments raised by the author. Thanks for understanding the approaches and the methods used for this paper.

Additional comments; we uploaded the figures for PACE tool and the output was ok.

References

[1]	Organization, W.H., Indicators for assessing infant and young child feeding practices: definitions and measurement methods. 2021.

[2]	Rutstein, S.O. and R. Winter, The effects of fertility behavior on child survival and child nutritional status: evidence from the demographic and health surveys, 2006 to 20122014: ICF International.
